# MASLD and Atherosclerosis in Patients with Type 2 Diabetes Mellitus: A Systematic Review

**DOI:** 10.3390/medicina62050919

**Published:** 2026-05-09

**Authors:** Cosmina-Theodora Vulpescu (Diaconu), Delia Reurean-Pintilei, Marius-Costin Chitu, Teodor Salmen, Anca Pantea Stoian, Cristian Guja

**Affiliations:** 1 Doctoral School, Carol Davila University of Medicine and Pharmacy, 020021 Bucharest, Romania; cosmina-theodora.diaconu@drd.umfcd.ro (C.-T.V.); marius-costin.chitu@drd.umfcd.ro (M.-C.C.); 2 Department of Medical-Surgical and Complementary Sciences, Faculty of Medicine and Biological Sciences, “Ștefan cel Mare” University, 720229 Suceava, Romania; delia.pintilei@usm.ro; 3 Department of Diabetes, Nutrition and Metabolic Diseases, Consultmed Medical Centre, 700544 Iasi, Romania; 4 Pitesti County Emergency Hospital, 110084 Pitesti, Romania; 5 Department of Diabetes, Nutrition and Metabolic Diseases, “Carol Davila” University of Medicine and Pharmacy, 020021 Bucharest, Romania; anca.stoian@umfcd.ro (A.P.S.); cristian.guja@hotmail.com (C.G.)

**Keywords:** type 2 diabetes, atherosclerosis, MASLD, NAFLD, carotid artery, femoral artery

## Abstract

*Background/Objectives*: Metabolic dysfunction-associated steatotic liver disease (MASLD) is highly prevalent among patients with type 2 diabetes (T2D) and has been increasingly recognized as a potential contributor to cardiovascular (CV) disease. However, the relationship between MASLD and subclinical/clinical atherosclerosis remains controversial, with inconsistent findings across imaging modalities and study populations. *Methods*: A systematic review was conducted according to PRISMA guidelines and registered in PROSPERO (CRD420261347480). Literature searches were performed across the PubMed, Scopus, and Web of Science library databases from 1 January 2016 to 27 March 2026, using the terms: “MASLD AND (type 2 diabetes OR type 2 diabetes mellitus OR T2DM) AND atherosclerotic plaque” for each of the three databases. Inclusion criteria comprised original full-text English-language studies, published in the last 10 years and conducted in adults, reporting data regarding the evaluation of atherosclerosis in patients with T2D and MASLD/NAFLD. Exclusion criteria are letters to the editor, expert opinions, case reports, conference or meeting abstracts, reviews, and redundant publications; having unclear or incomplete data; and being performed in vitro (cell cultures) or in animal models. The quality of included studies was assessed using the Newcastle–Ottawa Scale. *Results*: The included studies, predominantly cross-sectional and a single longitudinal study, as well as different modalities of evaluating atherosclerosis, showed heterogeneous findings. MASLD is associated with increased carotid plaque progression, including in lean individuals. Its relationship with carotid intima-media thickness (CIMT) is inconsistent across studies, with some reporting higher values and others finding no significant association after adjustment. Hepatic fibrosis appears more strongly linked to vascular aging than steatosis alone, with variability likely due to differences in study methods and populations. *Conclusions*: The presence of both MASLD and T2D may be associated with atherosclerosis across different stages, from subclinical changes to clinically manifest disease, particularly at more advanced stages such as plaque presence or progression, whereas its relationship with early markers like pulse wave velocity or CIMT remains inconsistent. Liver fibrosis may represent a stronger determinant of atherosclerosis than hepatic steatosis alone. Although the evidence base is limited and largely derived from a small number of predominantly cross-sectional studies, further standardized and prospective research is warranted to better define these relationships and evaluate CV risk stratification in patients with T2D.

## 1. Introduction

Metabolic dysfunction-associated steatotic liver disease (MASLD), previously termed non-alcoholic fatty liver disease (NAFLD), is defined as the hepatic steatosis occurring in the presence of cardiometabolic risk factors (obesity, type 2 diabetes (T2D), dyslipidemia, high blood pressure, etc.) and in the absence of other specific causes of liver fat accumulation. In recent years, it has become a major public health concern, affecting approximately 30% of adults worldwide, resulting in increased healthcare resource utilization and a substantial economic burden [1,2,3]. The prevalence of MASLD is significantly higher among patients with T2DM—approximately twice that of the general population—with European studies reporting the highest prevalence at around 68% [4,5].

Besides its epidemiological and economic burden, MASLD expresses a systemic disorder rather than an isolated hepatic condition. This fact is supported by the 2026 American Diabetes Association (ADA) Standards of Care, which integrate MASLD into the cardiometabolic continuum in patients with T2D, emphasizing structured screening, fibrosis-based risk stratification, and the implementation of integrated therapeutic strategies targeting both metabolic and cardiovascular (CV) risk factors [3].

The key mechanisms of this cardiometabolic state are represented by: insulin resistance (IR), chronic low-grade inflammation, pro-atherogenic lipid profile, microbiome alteration, and oxidative stress, which are also primary components in the development of T2D. Several shared pathophysiological pathways link MASLD and T2D to the development and progression of atherosclerosis, the leading cause of morbidity and mortality in this population.

Emerging data have linked MASLD with an increased risk of either subclinical or clinical manifestation of atherosclerosis, particularly in patients with T2D, including increased carotid intima-media thickness (IMT), plaque development, and major CV events [6,7]. Notably, this observation persists independently of traditional risk factors, suggesting that MASLD contributes to atherogenesis, rather than playing a passive role.

However, the available evidence remains heterogeneous, and the extent to which MASLD influences different stages and manifestations of atherosclerotic disease in patients with T2D is not yet clearly defined. In addition, current cardiovascular risk assessment strategies continue to rely primarily on conventional risk factors, which may not fully capture MASLD-related risk.

Therefore, the aim of this study was to conduct a systematic review of the existing literature evaluating the relationship between MASLD and atherosclerotic disease in adult patients with T2D, by assessing atherosclerotic plaque by characteristics and markers of its evolution.

## 2. Materials and Methods

A systematic review was performed according to the guidelines and recommendations from the Preferred Reporting Items for Systematic Reviews and Meta-Analysis Checklist (PRISMA). The protocol for this review has been registered with the identifier CRD420261347480.

### 2.1. Research Question and Search Strategy

An electronic search for relevant publications was performed using the PubMed, Scopus, and Web of Science library databases from 1 January 2016 to 27 March 2026. The following search strategy was used: “MASLD AND (type 2 diabetes OR type 2 diabetes mellitus OR T2DM) AND atherosclerotic plaque” for each of the three databases. In this search, 114 articles were found (5 from PubMed, 6 from Scopus, and 103 from Web of Science). We eliminated 9 duplicates and remained with 105 studies. After applying filters in each of the three databases for language (English), publication type (original articles), and date range (2016 to the date of the search), 44 articles remained, which underwent title screening, followed by abstract review by two independent reviewers. From the remaining 5 articles that were not about the subject of interest, they were eliminated, resulting in 39 articles that underwent initial title screening, followed by abstract review by two independent reviewers. After excluding the articles that did not meet the inclusion criteria, 3 articles remained for full assessment. We included 3 more articles identified when we searched the references of the relevant studies, leading to a total of 6 included articles.

The research question was framed using the Population, Intervention, Comparison, and Outcome (PICO) method. The population was represented by adult patients with T2D and MASLD/NAFLD elements, with any data about the presence, characteristics, severity, treatment, or future development of atherosclerosis. The outcome was defined by any characteristics of atherosclerosis in adult patients with T2D and elements of MASLD/NAFLD, with effect measured by percentage, confidence interval (CIs), odds, or relative risks and correlation levels. We chose both MASLD/NAFLD due to the fact that MASLD is a relatively recent term, which, during our period of interest, was preceded by NAFLD. However, although the discrepancy between NAFLD and MASLD appears minimal, MASLD incorporates explicit metabolic criteria that were not uniformly applied in NAFLD studies, which may introduce some heterogeneity. Accordingly, NAFLD-based evidence was interpreted within the MASLD framework while acknowledging these limitations.

### 2.2. Inclusion Criteria

The studies were included if they met the following criteria:

(1) published in English;
(2) original full-text cohort and cross-sectional studies;
(3) conducted on adult human populations;
(4) published in the last ten years;
(5) included patients with T2D and elements of NAFLD/MASLD and assessed atherosclerotic characteristics;
(6) sufficient data provided, such as appropriate 95% CIs, *p*-values, or correlations with various risk factors and markers of disease severity.

### 2.3. Exclusion Criteria

Studies were excluded if they

(1) were letters to the editor, expert opinions, editorials, case reports, conference, meeting abstracts, or reviews;
(2) were redundant publications, included duplicate or overlapping populations;
(3) provided unclear or incomplete data;
(4) include type 1 Diabetes (T1D), or patients with prediabetes, or with T2D without elements of NAFLD/MASLD;
(5) were conducted on in vitro (cell cultures) or in animal models.

### 2.4. Data Extraction

Two authors used a self-made data extraction table to individually evaluate and extract the following data for each included literature reference: the reported characteristic of atherosclerosis with their outcomes as 95% CIs or mean values. Any differences in opinion were settled through discussion or consultation with a third author.

### 2.5. Risk of Bias Assessment

Two reviewers independently assessed the quality of the studies using the Newcastle–Ottawa Scale (NOS), a star rating system that evaluates articles on selection, comparability, and outcome criteria [8]. Research papers rated with at least six stars as evaluated on the NOS are considered of moderate to high quality.

### 2.6. Strategy for Data Synthesis

The relatively small number of included studies (n = 6) reflects a limited body of literature that addresses atherosclerotic plaque characteristics and markers of its evolution in patients with T2D and NAFLD/MASLD in the last 10 years, alongside strict inclusion criteria focusing on the adult human population. Due to substantial heterogeneity between studies, a narrative synthesis was performed using text and tables to provide a descriptive summary and explanation of study characteristics and findings. Due to these facts, a meta-analysis was not performed, preventing meaningful pooling of results.

## 3. Results

**Figure 1:**
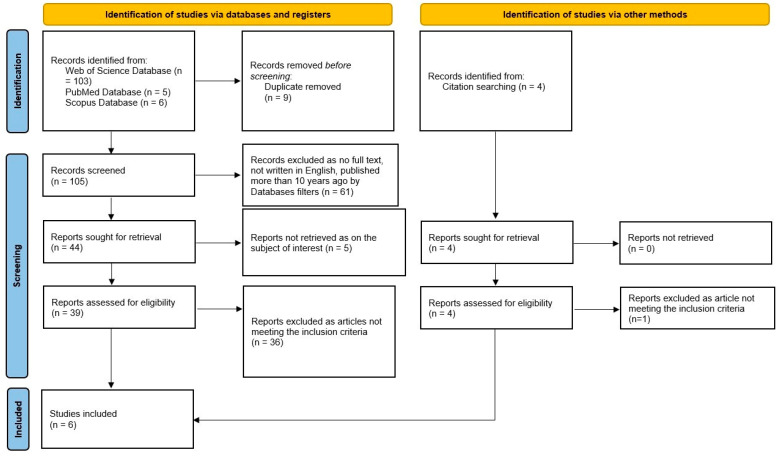
Flowchart of the study selection process.

The literature search from 27 March 2026 identified 114 records (103 from Web of Science Database, 5 from PubMed Database, and 6 from Scopus Database), which underwent the study selection process as summarized in Figure 1 (PRISMA 2020 flow diagram), which also details the identification, screening, eligibility, and inclusion stages.

9 duplicates were removed, leading to 105 records that underwent initial screening by applying filters for language (English), publication type (original articles), and date range (2016 to the date of the search) in each of the three databases.

Following the initial screening process that used the database’s predefined filters, we proceeded to evaluate the records sought for retrieval, and 5 more records were eliminated as not meeting the subject of interest domain. 39 records were assessed for eligibility by two independent reviewers, and 36 of them were excluded as not meeting the inclusion criteria. Any of the two reviewers’ disagreements were settled with a third reviewer. Alongside the 3 included studies, 4 records were identified from citation searching, and because none of them were retrieved and only one was excluded as not meeting the inclusion criteria, a total of six articles published between 2016 and 2026 were included for qualitative synthesis.

Research papers rated with at least six stars as evaluated on the NOS are considered of moderate to high quality and included in the Section 3, and the selection process is summarized in Table 1.

The characteristics and parameters of interest for the included studies for adult patients with T2D and diagnostic of MASLD/NAFLD, and any data about the presence, characteristics, severity, treatment, or future development of atherosclerosis are synthesized in Table 2 and Table 3.

## 4. Discussion

This systematic review synthesized current evidence regarding a possible association between the presence of MASLD and the development of atherosclerosis in adult patients with T2D. For the diagnosis of MASLD, abdominal ultrasound (US) or proton magnetic resonance spectroscopy ([1H]-MRS) was used, while to quantify fibrosis, non-invasive tests (NIT), such as the NAFLD Fibrosis Score (NFS), were used. The included studies assessed a range of markers of subclinical atherosclerosis, such as structural plaque-related parameters (presence, plaque progression, and distribution), arterial wall thickness measurements such as IMT (intima-media-thickness) and CIMT (Carotid intima-media thickness), and functional vascular indices including pulse wave velocity, vascular age, and carotid strain. These findings support a multifactorial model in which MASLD and T2D interact through shared mechanisms, including IR, chronic low-grade inflammation, lipid dysregulation, mitochondrial dysfunction, and endothelial impairment, to promote vascular injury and the development of atherosclerosis. However, the strength and consistency of these associations vary depending on study design, population characteristics, and the methodologies used to assess hepatic and vascular parameters.

### 4.1. MASLD and Atherosclerosis Through IR

The relationship between MASLD and atherosclerosis can be explained by shared pathophysiological mechanisms, with IR acting as a central driver.

IR increases lipolysis in adipose tissue, leading to elevated circulating free fatty acids (FFAs) that accumulate in the liver. This promotes hepatic triglyceride (TG) synthesis and overproduction of very-low-density lipoproteins (VLDL), driven by increased apo B100, apo CIII, and microsomal triglyceride transfer protein activity [15,16]. At the same time, impaired FFA oxidation and increased de novo lipogenesis further contribute to lipid accumulation, which in turn contributes to hepatic steatosis and systemic metabolic imbalance.

In addition to IR–driven lipid overload, the liver plays a primary role in lipid metabolism, and its metabolic dysfunction contributes to the development of an atherogenic lipid profile characterized by hypertriglyceridemia, increased low-density lipoprotein (LDL), and reduced high-density lipoprotein (HDL) levels. This dyslipidemia promotes lipid accumulation in both hepatic and extrahepatic tissues, including vascular beds, thereby contributing to plaque formation and increasing CV risk. Adipose tissue dysfunction further amplifies this process through increased FFA release and ectopic fat deposition, while alterations in adipokines—particularly reduced adiponectin levels—lead to impaired insulin sensitivity, enhanced inflammation, and endothelial dysfunction. Excess FFAs and toxic lipid intermediates induce oxidative stress, chronic low-grade inflammation, mitochondrial dysfunction, and endothelial injury, ultimately resulting in vascular injury and the development of atherosclerosis.

### 4.2. MASLD and Subclinical Atherosclerosis

Non-invasive imaging plays a primary role in the assessment of subclinical atherosclerosis in patients with MASLD and T2D, enabling early detection of plaque formation before the onset of CV disease. Carotid ultrasonography (US) is the most widely used modality, allowing evaluation of CIMT and, later, the presence of carotid plaques.

Among the studies included in this systematic review [9,10,11,12,13,14], the association between MASLD and CIMT was inconsistent. While some studies reported increased CIMT in patients with MASLD and T2D [11,12,13], only one longitudinal study reported an association between MASLD and both increased CIMT and carotid plaque progression over time [9].

These heterogeneous findings may reflect differences in study design, population characteristics, imaging techniques, and adjustment for metabolic confounders.

However, other studies, not included in this review, where hepatic steatosis assessed by computed tomography or [1H]-MRS was not associated with CIMT in populations with a high prevalence (82%) of T2D [17,18].

CIMT is widely recognized as an independent predictor of cardiovascular risk and is associated with future coronary and cerebrovascular events, even among asymptomatic individuals [19].

### 4.3. MASLD and Advanced Atherosclerosis

Among the studies included, a more consistent association was observed between MASLD and advanced atherosclerotic outcomes, particularly carotid artery plaque (CAP) presence and progression. Several studies reported an increased risk of CAP or its progression in patients with MASLD, after adjustment for confounding factors [9,10,13].

Longitudinal data, although limited, support this association. Kim et al. demonstrated that MASLD was associated with a higher risk of carotid plaque progression over time [9]. Similarly, Cho et al. reported significantly higher rates of CAP progression over a 7-year follow-up period in patients with NAFLD [13].

These findings suggest that MASLD may be more consistently associated with advanced structural vascular changes rather than early markers of atherosclerosis.

A potential role of disease severity is also suggested by findings indicating that hepatic fibrosis is associated with greater vascular impairment compared to steatosis alone, including increased CIMT and higher vascular age [11]. However, these observations are derived from a limited number of studies and should be interpreted with caution.

Even in patients without T2D, individuals with MASLD and higher Index for Liver Fibrosis (FIB-4) values (>1.3) demonstrated a significantly greater number of affected vascular territories and a larger plaque area, primarily involving the carotid artery [20]. Both FIB-4 and NFS have also been reported as independent predictors of carotid plaque presence after adjustment for conventional cardiovascular risk factors, whereas no significant associations were observed for femoral plaques [20]. This finding is consistent with previous reports suggesting a possible association between hepatic fibrosis and carotid atherosclerosis, although it should be interpreted with caution [21].

Evidence regarding the association between MASLD and carotid plaque remains heterogeneous. While several included studies demonstrated an association with plaque presence and progression, other studies from the literature suggest that NAFLD may be more strongly associated with carotid stenosis rather than plaque presence per se, with approximately a two-fold increased risk of significant stenosis after adjustment for conventional risk factors [18]. This variability may indicate that MASLD is associated with different stages or phenotypes of atherosclerosis, including vascular remodelling and luminal narrowing, although these findings should be interpreted cautiously.

Notably, this association has also been observed in lean individuals, with patients with lean MASLD exhibiting higher rates of plaque progression and increased cardiovascular risk compared to metabolically healthy lean individuals. However, these findings are primarily derived from literature and should be interpreted in the context of the limited and heterogeneous evidence base.

Although none of the included studies have evaluated coronary artery calcium (CAC), evidence from the literature suggests that individuals with NAFLD have an increased risk of CAC progression, independent of metabolic syndrome status [22,23]. Coronary artery calcium score (CACS), assessed by computed tomography, provides a quantitative measure of coronary atherosclerotic burden and is strongly associated with incident cardiovascular events, particularly in high-risk populations such as patients with T2D [24,25]. In patients with MASLD, CACS may be considered for further cardiovascular risk stratification when standard risk assessment tools are insufficient, although its routine use is not recommended based solely on the presence of MASLD [26]. In selected individuals with more severe MASLD and symptoms suggestive of underlying coronary artery disease, coronary computed tomography angiography may be considered because of its high sensitivity and specificity [27].

### 4.4. Hepatic Phenotype: Steatosis Versus Fibrosis

Among the studies included in this review, findings suggest that hepatic fibrosis may be associated with greater vascular impairment compared to steatosis alone, including increased CIMT and higher vascular age [11].

Importantly, longitudinal data indicate that hepatic fibrosis, defined as FIB-4 ≥  1.45, may represent an important determinant of atherosclerosis progression, with patients exhibiting advanced liver disease demonstrating significantly higher rates of carotid plaque progression over time. This relationship remains independent of traditional metabolic risk factors [28]. These findings are consistent with studies showing that higher fibrosis scores (e.g., FIB-4, NFS) are associated with increased plaque burden and vascular involvement [20].

### 4.5. MASLD, Atherosclerosis and Anthropometric Indices

Although anthropometric parameters were not consistently evaluated across the included studies, some evidence suggests that the association between MASLD and atherosclerosis is not solely dependent on obesity. In particular, observations from the included studies indicate that MASLD may be associated with plaque progression even in individuals without obesity [9,10].

A close association between MASLD and obesity has consistently been reported in the literature [29,30]. It has been posted that both general and central obesity are independently associated with the development of MASLD, with central obesity showing a stronger association than body mass index (BMI) alone [31]. Measures such as waist circumference and waist-to-hip ratio were more strongly linked to MASLD risk than overall adiposity, suggesting that visceral fat plays a key role in disease pathogenesis. These findings support the concept that metabolic dysfunction, rather than total body weight, is the primary driver of hepatic steatosis. Furthermore, central obesity is closely associated with IR, dyslipidemia, and adipokine secretion, all of which contribute to systemic inflammation and may promote the development of atherosclerosis [32,33].

Additional data suggest that individuals with lean NAFLD exhibited significantly higher atherosclerotic CV disease (ASCVD) risk scores compared to those with obese NAFLD, indicating that the absence of obesity does not confer protection against CV risk. This association remained consistent even when obesity was defined using a BMI ≥ 30 kg/m^2^. After multivariable adjustment, lean subjects with NAFLD demonstrated a higher CV risk than both obese individuals with and without NAFLD. Notably, the highest ASCVD risk was observed in lean individuals with significant liver fibrosis, followed by obese individuals with fibrosis, underscoring the importance of disease severity over adiposity in determining CV outcomes [34].

### 4.6. Therapies That Decrease the Atherosclerotic Risk

Recent evidence suggests that glucagon-like peptide-1 receptor agonist (GLP-1 RA) may exert beneficial effects beyond glycemic management in patients with T2DM, including improvements in metabolic parameters and markers of hepatic injury associated with MASLD (reducing FIB-4). These effects are accompanied by the reductions in CV risk factors, supporting the concept of a shared cardiometabolic pathway linking liver dysfunction and the presence of atherosclerosis [35]. Although this study is limited by a small sample size and a lack of a control study design. These findings are consistent with the associations identified in this review, particularly the link between advanced disease of MASLD and increased atherosclerotic burden. This highlights the potential relevance of integrated therapeutic strategies targeting both hepatic and vascular risk, and suggests that MASLD status may be an important consideration in cardiovascular risk stratification and treatment decisions in patients with T2D [35].

Furthermore, the 2026 American Diabetes Association (ADA) Standards of Care have incorporated MASLD as a specific factor in therapeutic decision-making, with GLP-1RA preferred for glycemic management in patients with steatohepatitis (MASH) or with high-risk fibrosis, alongside weight reduction as a cornerstone of treatment [36].

### 4.7. Atherosclerosis in Patients with MASLD and T1D

In adults with T1D, the prevalence of MASLD/NAFLD ranges from 4.7 to 52% [37,38,39]. This huge discrepancy may be due to differences in the methods used to evaluate steatosis; the smallest prevalence was seen with the use of MR imaging, while the highest one was identified by abdominal US.

Data from a small study that included 128 patients with T1D, of whom 6 patients had hepatic steatosis, show no association with increased IMT or prior CV events, suggesting that hepatic fat accumulation may not independently contribute to sub-clinical atherosclerosis. Emerging data postulate that age remains the dominant determinant of vascular changes [38]. In contrast, other studies have reported an association between hepatic steatosis and markers of subclinical atherosclerosis in T1D. Some findings suggest that while NAFLD in T1D may be associated with early vascular changes (IMT), more advanced atherosclerotic markers (CACS, plaques) appear to be more closely related to liver enzyme elevations rather than steatosis per se [40].

### 4.8. Interpretation of Heterogeneity

An important source of heterogeneity across the included studies is the type of vascular abnormality assessed, as the selected articles do not evaluate atherosclerosis at the same stage of vascular involvement. Some studies focused on baseline structural burden, such as carotid plaque presence and number, or increased IMT/CIMT, thereby reflecting already established subclinical atherosclerosis. In contrast, longitudinal studies assessed plaque progression over time, offering insight into the dynamic evolution of vascular disease rather than its cross-sectional presence alone. Other investigations evaluated functional vascular changes, including pulse wave velocity, carotid strain, and vascular age, which may represent earlier manifestations of vascular dysfunction preceding overt plaque formation. This variation in vascular endpoints is clinically relevant, as it suggests that MASLD in patients with T2D may be associated not only with existing plaque burden, but also with progressive arterial remodelling and early vascular impairment. Consequently, differences in the type of vascular marker assessed may partly explain the variability of findings across studies and should be considered when interpreting the strength and consistency of the observed associations.

### 4.9. Strengths and Limitations

This review has several noteworthy strengths. It follows a systematic methodological approach based on PRISMA guidelines and independent risk of bias assessment, integration of both subclinical and clinical atherosclerosis with the evaluation of the presence of MASLD in patients with T2D, which enhances the translational value of the findings for risk stratification in those patients. Also, this review allows for a clear identification of knowledge gaps and future research directions in the field.

However, several limitations should be acknowledged, such as heterogeneity in the evaluation of hepatic steatosis assessment methods, as well as the definition applied for the diagnosis of MASLD/NAFLD, which limits direct comparability between studies; the search strategy may seem too narrow regarding the ange of the plaque-based terminology and may miss relevant studies about CIMT, intima-media thickness, arterial stiffness, pulse wave velocity, coronary artery calcium, vascular calcification, endothelial dysfunction, carotid atherosclerosis, and subclinical atherosclerosis. Atherosclerosis assessments were often conducted in relatively small cohorts, many of them from Asia, which may limit generalizability. Further limitations include heterogeneity in imaging modalities and restriction to English-language publications, potentially introducing selection bias. Furthermore, most included studies were cross-sectional, with only a limited number of longitudinal cohort studies, thereby restricting the ability to establish causal relationships.

### 4.10. Future Perspectives

Future research should focus on longitudinal studies, especially randomized controlled trials, to better define the additional CV risk for patients with T2DM that associate MASLD. Additionally, there is a need to develop dedicated CV risk scores based on T2DM population that integrate the fibrosis evaluation.

Given the persistently elevated CV risk in patients with T2DM, early detection of subclinical vascular changes and intensive management of modifiable risk factors are essential to prevent established CV disease. To date, risk stratification has largely relied on traditional risk factors; however, integrating emerging diseases such as MASLD may improve the identification of individuals at the highest risk.

For our future projects, we plan to expand the search terms to encompass a wider range of vascular outcomes, not only the plaque—an important endpoint, but with markers such as CAC and other indices of vascular function and subclinical disease. It remains a question whether certain vascular territories (carotid) are affected, whereas others (femoral) are not, requiring dedicated mechanistic studies, particularly across patients with T2DM and MASLD.

## 5. Conclusions

The presence of MASLD in patients with T2D appears to be associated with an increased risk of atherosclerosis and CV disease, with more consistent findings observed at the moment of advanced stages, such as carotid plaque presence. In contrast, its relationship with early markers of atherosclerosis, such as CIMT, remains inconsistent across studies, likely reflecting differences in study design, imaging modalities, and adjustment for metabolic confounders.

Importantly, liver fibrosis appears to be more strongly associated with the prognosis of vascular disease and may represent a major determinant of CV risk beyond steatosis alone. These findings support the concept that MASLD is not solely a hepatic condition but part of a systemic cardiometabolic disorder.

From a clinical perspective, many patients with MASLD also have T2D. Given that CV risk remains high, early detection of subclinical vascular involvement and intensive medical risk factor control are essential in the prevention of manifest CV disease.

However, these considerations should be interpreted with caution in light of the limited number of included studies and their heterogeneity. Future research should focus on well-designed prospective studies and randomized controlled trials to clarify the causal relationship between MASLD and atherosclerosis, particularly for patients with T2D, and to determine whether incorporating subclinical markers of atherosclerosis into CV risk stratification models improves clinical outcomes.

## Figures and Tables

**Table 1:** Newcastle–Ottawa Scale analysis of the included studies included [9,10,11,12,13,14].

Author (Reference)	Selection				Comparability	Outcome			Total Score	Quality
	Representativeness of the Exposed Cohort	Selection of the Non-Exposed Cohort	Ascertainment of Exposure	Demonstration That Outcome of Interest Was Not Present at Start of Study	Comparability of Cohorts Based on the Design or Analysis	Assessment of Outcome	Was Follow-Up Long Enough for Outcomes to Occur	Adequacy of Follow-Up of Cohorts		
Kim, Y. et al., 2025 [9]	+	−	+	+	+	+	+	+	7	Very good
Pan, J et al., 2025 [10]	+	−	+	+	+	+	−	+	6	Good
Coutinho, D.A.A. et al., 2025 [11]	+	−	+	+	+	+	−	+	6	Good
Zhu, Y et al., 2025 [12]	+	−	+	+	+	+	−	+	6	Good
Cho, Y et al., 2023 [13]	+	−	+	+	+	+	−	+	6	Good
Li, Z et al., 2022 [14]	+	−	+	+	+	+	−	+	6	Good

“+” indicates that the article meets the criteria mentioned above; “−” indicates that the article does not meet the abovementioned criteria.

**Table 2:** Included studies characteristics.

First Author, Publication Year	Location	Sample	Groups	MASLD/NAFLD Diagnostic Criteria
Kim, Y. et al., 2025 [9]	Seoul	828	G1—197 patients (23.8%) lean without MASLD G2—57 (6.9%) lean with MASLD G3—177 (21.4%) non-lean without MASLD G4—397 (47.9%) non-lean with MASLD	MASLD was confirmed by abdominal US lean body mass was defined as a BMI < 23 kg/m^2^
Pan, J et al., 2025 [10]	China	8644	G1—non-hepatic steatosis G2—dysglycemia-MASLD G3—overweight-MASLD G4—lean-MASLD G5—other hepatic steatosis.	US + ≥1 cardio-metabolic risk factors
Coutinho, D.A.A. et al., 2025 [11]	Brazilia	114	Steatosis—99 participants (86.8%) Fibrosis—31 participants (27.2%)	US and liver elastography
		85	The T2D subgroup that was analysed included 27 (31.8%) patients with fibrosis versus 58 (68.2%) without fibrosis	
Zhu, Y et al., 2025 [12],	NR	240	120 new T2D 60 pre-diabetes mellitus 60 healthy controls	Liver fat content assessed using proton magnetic resonance spectroscopy ([1H]-MRS)
Cho, Y et al., 2023 [13]	Seoul	852	no NAFLD/ sarcopenia NAFLD only sarcopenia only NAFLD with sarcopenia	Abdominal US
Li, Z et al., 2022 [14]	China	98	Group A—35 individuals without NAFLD Group B—33 individuals with moderate NAFLD Group C—30 individuals with moderate to severe NAFLD	NAFLD diagnosed by liver US

MASLD—metabolically associated steatotic liver disease; NAFLD—non-alcoholic fatty-liver disease; liver disease; US—ultrasonography; BMI—body mass index.

**Table 3:** Included studies’ parameters of interest.

First Author, Publication Year	Parameter	Dimension	Statistical Power
Kim, Y. et al., 2025 [9]	Progression of carotid atherosclerosis according to BMI and hepatic status	G1—29.4% G2—47.4% G3—38.4% G4—40.6%	*p* = 0.025
	CAP progression	G2 versus G1	OR 2.16 95% CI (1.18,3.94), *p* = 0.013
			aOR 2.57, 95% CI 1.33–4.97; *p* = 0.005
		G3 versus G1	OR 1.50 95% CI (0.97, 2.30), *p* = 0.067
			aOR 1.62, 95% CI 1.00–2.63; *p* = 0.051
		G4 versus G1	OR 1.64 95% CI (1.13, 2.36), *p* = 0.008
			aOR 1.59, 95% CI 1.05–2.42; *p* = 0.029
Pan, J et al., 2025 [10]	CAP prevalence	G2	26.28%
		G4	18.55%
		G3	14.39%
	Risk of CAP	G2	OR 1.599 (95% CI 1.348, 1.896)
		under 45 years old G2 versus G1	ORs 2.393 (95% CI 1.660, 3.416)
		under 45 years old G4 versus G1	ORs 2.724 (95% CI 1.002, 6.221),
Coutinho, D.A.A. et al., 2025 [11]	CAP frequency	33 participants (28.9%)	no significant difference between groups
	CIMT	Higher in fibrosis versus steatosis group	
	vascular age	Higher in fibrosis versus steatosis group	
	CIMT	0.742 mm versus 0.653 mm	*p* < 0.05
	Vascular age	exceeded chronological age by 9 years	*p* < 0.05
Zhu, Y et al., 2025 [12]	Liver fat content	14.57% ± 6.49% versus 11.44% ± 5.62% versus 5.73% ± 4.08%	*p* < 0.0001
	CIMT	Elevated in new T2D versus prediabetes mellitus versus healthy control	*p* < 0.05
	pulse wave velocity at the beginning of systole	Elevated in new T2D versus prediabetes mellitus versus healthy control	*p* < 0.05
	pulse wave velocity at the end of systole	Elevated in new T2D versus prediabetes mellitus versus healthy control	*p* < 0.05
Cho, Y et al., 2023 [13]	CIMT at baseline	0.76 ± 0.15 versus 0.77 ± 0.15 versus 0.74 ± 0.14 versus 0.79 ± 0.14	*p* = 0.042
	CAP at baseline	115 (34.8%) versus 107 (32.1%) versus 19 (28.8%) versus 46 (37.4%)	*p* = 0.567
	CAP progression at 7 years of follow-up	86 (26.1%) versus 109 (32.7%) versus 35 (53.0%) versus 61 (49.6%)	*p* < 0.001
Li, Z et al., 2022 [14]	CIMT (mm)	0.83 ± 0.19 versus 0.64 ± 0.12 for Group C versus Group A	*p* < 0.05
		0.83 ± 0.19 versus 0.66 ± 0.14 for Group C versus Group B	*p* < 0.05
	circumferential strain of carotid artery	6.99 ± 1.91 versus 8.57 ± 1.90 for Group B versus Group A	*p* < 0.05
		5.43 ± 1.30 versus 8.57 ± 1.90 for Group C versus Group A	*p* < 0.05
		5.43 ± 1.30 versus 6.99 ± 1.91 for Group C versus Group B	*p* < 0.05

OR—odds ratio; aOR—adjusted odds ratio; CI—confidence interval; CIMT—carotid intima-media thickness; CAP—carotid artery plaque; T2D—type 2 diabetes.

## Data Availability

All data are contained within this manuscript.

## Abbreviations

The following abbreviations are used in this manuscript:

CV	Cardiovascular
MASLD	Metabolic Dysfunction-Associated Steatotic Liver Disease
NAFLD	Non-Alcoholic Fatty Liver Disease
T1D	Type 1 Diabetes
T2D	Type 2 Diabetes
CIMT	Carotid intima-media thickness
CAC	Coronary artery calcium
FIB-4	Index for Liver Fibrosis
CAP	Carotid artery plaque
BMI	Body mass index
ASCVD	Atherosclerotic CV disease
GLP-1 RA	Glucagon-like peptide-1 Receptor Agonists
US	abdominal ultrasound
[1H]-MRS	proton magnetic resonance spectroscopy

## References

[1] Younossi Z.M., Golabi P., Paik J.M., Henry A., Van Dongen C., Henry L. (2023). The global epidemiology of nonalcoholic fatty liver disease (NAFLD) and nonalcoholic steatohepatitis (NASH): A systematic review. Hepatology.

[2] International Diabetes Federation (2025). IDF Diabetes Atlas.

[3] American Diabetes Association Professional Practice Committee (2026). Comprehensive Medical Evaluation and Assessment of Comorbidities: Standards of Care in Diabetes—2026. Diabetes Care.

[4] Younossi Z.M., Golabi P., Price J.K., Owrangi S., Gundu-Rao N., Satchi R., Paik J.M. (2024). The global epidemiology of nonalcoholic fatty liver disease and nonalcoholic steatohepatitis among patients with type 2 diabetes. Clin. Gastroenterol. Hepatol..

[5] Cusi K., Abdelmalek M.F., Apovian C.M., Balapattabi K., Bannuru R.R., Barb D., Bardsley J.K., Beverly E.A., Corbin K.D., ElSayed N.A. (2025). Metabolic Dysfunction-Associated Steatotic Liver Disease (MASLD) in People with Diabetes: The Need for Screening and Early Intervention—A Consensus Report of the American Diabetes Association. Diabetes Care.

[6] Aussy C., Aubin A., Loomba R. (2021). The relationship between type 2 diabetes, NAFLD, and cardiovascular risk. Curr. Diab. Rep..

[7] Targher G., Corey K.E., Byrne C.D. (2021). NAFLD and cardiovascular and cardiac diseases: Factors influencing risk, prediction and treatment. Diabetes Metab..

[8] Wells G.A., Shea B., O’Connell D., Pereson J., Welch V., Losos M., Tugwell P. (2011). The Newcastle-Ottawa Scale (N.O.S.) for Assessing the Quality of Nonrandomized Studies in Meta-Analyses.

[9] Kim Y., Cho Y., Lee Y.H., Seo D.H., Ahn S.H., Hong S., Kim S.H. (2025). Association of lean metabolic dysfunction-associated steatotic liver disease with carotid plaque progression in patients with type 2 diabetes mellitus. J. Diabetes Investig..

[10] Pan J., Zeng H., Song Y., Zhang X., Wang Z., Tang L., Xie B., Peng R., Zhou Y., Liu B. (2025). Associations between MASLD phenotypes and the risk of carotid artery plaque: A cross-sectional study among railway workers. Acta Diabetol..

[11] Coutinho D.A.A., Godinho J.R., Moura R.C., Oliveira R.M., Passos H.F., Mendes J.P.F., Andrade Junior C.R.M., Coca-Velarde L.G., Saad M.A.N., Flores P.P. (2025). Relationship between Liver Fibrosis Due to Metabolic Dysfunction-Associated Steatotic Liver Disease and Subclinical Atherosclerosis. Relação entre Fibrose Hepática Decorrente da Doença Hepática Gordurosa Associada à Disfunção Metabólica e Aterosclerose Subclínica. Arq. Bras. Cardiol..

[12] Zhu Y., Wang Y.S., Zhang G.P., Ma F., Xia J.X., Lian H., Cao Y.H., Zhang R., Ye J., Dai W. (2025). Association of Liver Fat Content With Carotid Endothelial Function in Patients With Newly Diagnosed Type 2 Diabetes Mellitus and Pre-Diabetes Mellitus. Angiology.

[13] Cho Y., Park H.S., Huh B.W., Lee Y.H., Seo S.H., Seo D.H., Ahn S.H., Hong S., Kim S.H. (2023). Non-Alcoholic Fatty Liver Disease with Sarcopenia and Carotid Plaque Progression Risk in Patients with Type 2 Diabetes Mellitus. Diabetes Metab. J..

[14] Li Z., Mao X., Cui X., Yu T., Zhang M., Li X., Li G. (2022). Evaluate the elasticity of carotid artery in the type 2 diabetes mellitus patients with nonalcoholic fatty liver disease by two-dimensional strain imaging. Medicine.

[15] Taghibiglou C., Carpentier A., Van Iderstine S.C., Chen B., Rudy D., Aiton A., Lewis G.F., Adeli K. (2000). Mechanisms of hepatic very low density lipoprotein overproduction in insulin resistance: Evidence for enhanced lipoprotein assembly, reduced intracellular ApoB degradation, and increased microsomal triglyceride transfer protein in a fructose-fed hamster model. J. Biol. Chem..

[16] Chen M., Breslow J.L., Li W., Leff T. (1994). Transcriptional regulation of the apoC-III gene by insulin in diabetic mice: Correlation with changes in plasma triglyceride levels. J. Lipid Res..

[17] McKimmie R.L., Daniel K.R., Carr J.J., Bowden D.W., Freedman B.I., Register T.C., Hsu F.C., Lohman K.K., Weinberg R.B., Wagenknecht L.E. (2008). Hepatic steatosis and subclinical cardiovascular disease in a cohort enriched for type 2 diabetes: The Diabetes Heart Study. Am. J. Gastroenterol..

[18] Guo Y.C., Zhou Y., Gao X., Yao Y., Geng B., Cui Q.H., Yang J.C., Hu H.P. (2018). Association between Nonalcoholic Fatty Liver Disease and Carotid Artery Disease in a Community-Based Chinese Population: A Cross-Sectional Study. Chin. Med. J..

[19] Greenland P., Alpert J.S., Beller G.A., Benjamin E.J., Budoff M.J., Fayad Z.A., Foster E., Hlatky M.A., Hodgson J.M., Kushner F.G. (2010). 2010 ACCF/AHA guideline for assessment of cardiovascular risk in asymptomatic adults. J. Am. Coll. Cardiol..

[20] León-Mengíbar J., Malagón M.M., Bermúdez-López M., Valdivielso J.M., Pamplona R., Torres G., Mauricio D., Castro E., Fernández E., Caixàs A. (2026). Association of estimated liver fibrosis with carotid but not femoral atherosclerotic burden: The ILERVAS cohort. Front. Endocrinol..

[21] Nyberg L.M., Cheetham T.C., Patton H.M., Yang S.J., Chiang K.M., Caparosa S.L., Stern J.A., Nyberg A.H. (2020). The Natural History of NAFLD, a Community-Based Study at a Large Health Care Delivery System in the United States. Hepatol. Commun..

[22] Cho Y.K., Kang Y.M., Yoo J.H., Lee J., Lee S.E., Yang D.H., Kang J.-W., Park J.-Y., Jung C.H., Kim H.-K. (2018). The impact of non-alcoholic fatty liver disease and metabolic syndrome on the progression of coronary artery calcification. Sci. Rep..

[23] Stols-Gonçalves D., Hovingh G.K., Nieuwdorp M., Holleboom A.G. (2019). NAFLD and atherosclerosis: Two sides of the same dysmetabolic coin?. Trends Endocrinol. Metab..

[24] Denissen S.J.A.M., van der Aalst C.M., Vonder M., Gratama J.W.C., Adriaansen H.J., Kuijpers D., Lennep J.E.R.V., Vliegenthart R., van der Harst P., Braam R.L. (2021). Screening for coronary artery calcium in a high-risk population: The ROBINSCA trial. Eur. J. Prev. Cardiol..

[25] Marx N., Federici M., Schütt K., Müller-Wieland D., A Ajjan R., Antunes M.J., Christodorescu R.M., Crawford C., Di Angelantonio E., Eliasson B. (2023). 2023 ESC guidelines for the management of cardiovascular disease in patients with diabetes. Eur. Heart J..

[26] Chew N.W., Mehta A., Goh R.S.J., Zhang A., Chen Y., Chong B., Chew H.S.J., Shabbir A., Brown A., Dimitriadis G.K. (2025). Cardiovascular-liver-metabolic health: Recommendations in screening, diagnosis, and management of metabolic dysfunction-associated steatotic liver disease in cardiovascular disease via modified Delphi approach. Circulation.

[27] Ngam P.I., Ong C.C., Chai P., Wong S.S., Liang C.R., Teo L.L.S. (2020). Computed tomography coronary angiography—Past, present and future. Singap. Med. J..

[28] Lee H.-H., Cho Y., Choi Y.J., Huh B.W., Lee B.-W., Kang E.S., Park S.W., Cha B.-S., Lee E.J., Lee Y.-H. (2020). Non-alcoholic steatohepatitis and progression of carotid atherosclerosis in patients with type 2 diabetes: A Korean cohort study. Cardiovasc. Diabetol..

[29] Yoo J.-J., Kim W., Kim M.Y., Jun D.W., Kim S.G., Yeon J.-E., Lee J.W., Cho Y.K., Park S.H., Sohn J.H. (2019). Recent research trends and updates on nonalcoholic fatty liver disease. Clin. Mol. Hepatol..

[30] Polyzos S.A., Kountouras J., Mantzoros C.S. (2019). Obesity and nonalcoholic fatty liver disease: From pathophysiology to therapeutics. Metabolism.

[31] Pang Q., Zhang J.Y., Song S.D., Qu K., Xu X.S., Liu S.S., Liu C. (2015). Central obesity and nonalcoholic fatty liver disease risk after adjusting for body mass index. World J. Gastroenterol..

[32] Schäffler A., Schölmerich J., Büchler C. (2005). Mechanisms of disease: Adipocytokines and visceral adipose tissue—Emerging role in nonalcoholic fatty liver disease. Nat. Clin. Pract. Gastroenterol. Hepatol..

[33] Buechler C., Wanninger J., Neumeier M. (2011). Adiponectin, a key adipokine in obesity-related liver diseases. World J. Gastroenterol..

[34] Kim Y., Han E., Lee J.S., Lee H.W., Kim B.K., Kim M.K., Kim H.S., Park J.Y., Kim D.Y., Ahn S.H. (2022). Cardiovascular risk is elevated in lean subjects with nonalcoholic fatty liver disease. Gut Liver.

[35] Hachuła M., Kosowski M., Basiak M., Okopień B. (2023). Does Therapy with Glucagon-like Peptide 1 Receptor Agonists Have an Effect on Biochemical Markers of Metabolic-Dysfunction-Associated Steatotic Liver Disease (MASLD)? Pleiotropic Metabolic Effect of Novel Antidiabetic Drugs in Patients with Diabetes-Interventional Study. Pharmaceuticals.

[36] American Diabetes Association Professional Practice Committee (2026). Pharmacologic approaches to glycemic treatment: Standards of care in diabetes—2026. Diabetes Care.

[37] Corbin K.D., Driscoll K.A., Pratley R.E., Smith S.R., Maahs D.M., Mayer-Davis E.J. (2018). Obesity in type 1 diabetes: Pathophysiology, clinical impact, and mechanisms. Endocr. Rev..

[38] Petit J., Pedro L., Guiu B., Duvillard L., Bouillet B., Jooste V., Habchi M., Crevisy E., Fourmont C., Buffier P. (2015). Type 1 diabetes is not associated with an increased prevalence of hepatic steatosis. Diabet. Med..

[39] Targher G., Pichiri I., Zoppini G., Trombetta M., Bonora E. (2012). Increased prevalence of chronic kidney disease in patients with type 1 diabetes and non-alcoholic fatty liver. Diabet. Med..

[40] Serra-Planas E., Aguilera E., Castro L., Rodríguez R., Salinas I., Lucas A., Joaquín C., Puig R., Mauricio D., Puig-Domingo M. (2017). Low prevalence of non-alcoholic fatty liver disease in patients with type 1 diabetes is associated with decreased subclinical cardiovascular disease. J. Diabetes.

